# Wave generation and transmission in multi-scale complex media and structured metamaterials (part 2)

**DOI:** 10.1098/rsta.2022.0224

**Published:** 2022-11-28

**Authors:** Alexander B. Movchan, Gennady Mishuris, Federico Sabina

**Keywords:** waves, structured media, mathematical modelling, mechanics of solids and fluids

This is part 2 of the theme issue on ‘Wave generation and transmission in multi-scale complex media and structured metamaterials’. It is focused on the analysis of wave scattering and wave localization in complex media, including acoustic and elastic waves, vibrations in viscoelastic media, and pulsating flows associated with waves in fluids, with an interesting range of industrial applications as well as ideas linked to biomechanics and the mechanics of metamaterials. The volume is logically connected to part 1 of the theme issue, and the two parts are mutually complementary in their presentation of the state of the art in the areas of mathematical modelling, experimental assessment and industrial applications of wave generation and control, in the context of the dynamic response of multi-scale waveguides and a new generation of metamaterials. We would like to pay tribute to our colleague Dr Rabindra Kumar Bhattacharyya, who sadly passed away on 23 May 2021 and whose ideas were inspirational in the preparation of the theme issue.

The mathematical modelling of waves in multi-scale media is so incredibly fascinating and attractive—no wonder that many researchers and industrial scientists publish on this topic. Although some of the problems may appear to be classical and well studied, detailed mathematical analysis often brings new results. The Helmholtz oscillator formulation, which is widely regarded as a textbook problem, is in fact so rich that an advanced asymptotic analysis will reveal novel features in the structure of the scattered fields for special classes of scatterers and incident waves.

If many oscillators are assembled into a periodic system or a large cluster with patterns of periodicity, then the combined dynamic response of such a system becomes very interesting, with features such as dynamic anisotropy and standing waves of different shapes and forms. The mathematical and experimental studies of such multi-scale systems have led to the very popular concept of dynamic metamaterials.

A well-known phenomenon in mechanics is fracture, when an elastic solid is damaged by a crack, which may grow slowly or advance dynamically. If the master medium is a structured solid (for example, an elastic lattice in the simplest case), the waves in such a medium are dispersive, which provides interesting insights into the phenomenon of dynamic crack propagation. Although such a problem appears to be extremely difficult for analytical and numerical studies, elegant mathematical ideas can be used to reduce the study of a semi-infinite crack, propagating through a lattice at a given speed, to the analysis of a functional equation of Wiener–Hopf type. The kernel function of such an equation is linked to the Fourier transform of the lattice Green’s function. One can graph the dispersion curves of waves in the ambient lattice together with the line representing the motion of the crack. When this line is tangent to a dispersion curve, a resonance occurs, which may be viewed as a frontal wave. When a lattice incorporates built-in resonators, the effects of negative inertia (associated with phase shifts) within the ambient lattice significantly change the dynamics of the advancing crack. A notion of ‘dynamic fault’ is also used to characterize a crack advancing through a structured medium, and the analysis of the Wiener–Hopf functional equations can be successfully applied to study failure of structured waveguides. Practical applications include designs and studies of engineering bridge structures with periodically distributed supporting sub-structures. One well-known example is linked to the failure of the San Saba bridge in Texas in 2013, where the propagating failure wave was observed during the collapse.

The Floquet–Bloch theoretical framework has proved to be efficient in a wide range of models of wave propagation through periodic waveguides. However, if a perturbation is introduced in the structure, then localized waveforms, representing defect modes, may be observed, which may require additional analysis and an alternative mathematical approach. In time-harmonic regimes, such defect modes may also be associated with negative inertia. This is especially relevant to cases where multi-scale resonators are embedded into the structure of the waveguide and a phase shift occurs in vibrations of the master structure and of the embedded resonators.

Control of waves and methods of reducing scattering are of high importance due to strong interest from industry as well as fascinating modelling approaches. The notion of cloaking is common nowadays, but the idea of transformation cloaking goes back more than six decades to the work of Lev Dolin. In recent years, the concepts of invisibility cloaking have been developed for electromagnetic and acoustic waves as well as for vector problems of mathematical elasticity. One important class is related to active cloaking: several active devices generate auxiliary waveforms, which ‘cancel’ the effect of the incident wave. In acoustics, active noise-cancelling technology has already been embraced by Bose and other manufacturers. However, active cloaking in the control of vibrations of elastic flexural plates poses interesting challenges: a new idea that leads to new methodology is based on the use of evanescent devices, which produce localized waveforms in order to shield from an incident wave a desired area within the plate.

Both parts of the theme issue aim to bring together the fields of applied mathematics, solid mechanics, physics, structural and mechanical engineering, and environmental science to demonstrate the state of the art in the fascinating field of wave generation, control and transmission in structured waveguides as well as multi-physics applications.

## Data accessibility

This article has no additional data.

## Authors' contributions

A.B.M.: writing—review and editing; G.M.: writing—review and editing; F.S.: writing—review and editing.

All authors gave final approval for publication and agreed to be held accountable for the work performed therein.

## Conflict of interest declaration

This theme issue was put together by the Guest Editor team under supervision from the journal’s Editorial staff, following the Royal Society’s ethical codes and best-practice guidelines. The Guest Editor team invited contributions and handled the review process. Individual Guest Editors were not involved in assessing papers where they had a personal, professional or financial conflict of interest with the authors or the research described. Independent reviewers assessed all papers. Invitation to contribute did not guarantee inclusion.

## Funding

No funding has been received for this article.

